# Postnatal development of electrophysiological and morphological properties in layer 2/3 and layer 5 pyramidal neurons in the mouse primary visual cortex

**DOI:** 10.1093/cercor/bhac467

**Published:** 2022-12-01

**Authors:** Natalja Ciganok-Hückels, Kevin Jehasse, Lena Kricsfalussy-Hrabár, Mira Ritter, Thomas Rüland, Björn M Kampa

**Affiliations:** Systems Neurophysiology, Institute of Zoology, RWTH Aachen University, 52074 Aachen, Germany; Research Training Group 2416 MultiSenses-MultiScales, RWTH Aachen University, 52074 Aachen, Germany; Systems Neurophysiology, Institute of Zoology, RWTH Aachen University, 52074 Aachen, Germany; Systems Neurophysiology, Institute of Zoology, RWTH Aachen University, 52074 Aachen, Germany; Systems Neurophysiology, Institute of Zoology, RWTH Aachen University, 52074 Aachen, Germany; Systems Neurophysiology, Institute of Zoology, RWTH Aachen University, 52074 Aachen, Germany; Research Training Group 2416 MultiSenses-MultiScales, RWTH Aachen University, 52074 Aachen, Germany; Institute for Biological Information Processing (IBI-1), Forschungszentrum Jülich, 52428 Jülich, Germany; Systems Neurophysiology, Institute of Zoology, RWTH Aachen University, 52074 Aachen, Germany; Research Training Group 2416 MultiSenses-MultiScales, RWTH Aachen University, 52074 Aachen, Germany; JARA BRAIN, Institute of Neuroscience and Medicine (INM-10), Forschungszentrum Jülich, 52428 Jülich, Germany

**Keywords:** development, mouse, visual cortex, dendrites, electrophysiology

## Abstract

Eye-opening is a critical point for laminar maturation of pyramidal neurons (PNs) in primary visual cortex. Knowing both the intrinsic properties and morphology of PNs from the visual cortex during development is crucial to contextualize the integration of visual inputs at different age stages. Few studies have reported changes in intrinsic excitability in these neurons but were restricted to only one layer or one stage of cortical development. Here, we used in vitro whole-cell patch-clamp to investigate the developmental impact on electrophysiological properties of layer 2/3 and layer 5 PNs in mouse visual cortex. Additionally, we evaluated the morphological changes before and after eye-opening and compared these in adult mice. Overall, we found a decrease in intrinsic excitability in both layers after eye-opening which remained stable between juvenile and adult mice. The basal dendritic length increased in layer 5 neurons, whereas spine density increased in layer 2/3 neurons after eye-opening. These data show increased number of synapses after onset of sensory input paralleled with a reduced excitability, presumably as homeostatic mechanism. Altogether, we provide a database of the properties of PNs in mouse visual cortex by considering the layer- and time-specific changes of these neurons during sensory development.

## Introduction

During developmental processes, many changes occur in the cortical structures of the brain. Especially, the cortex of rodents undergoes rapid and dramatic changes in the first postnatal month (Lendvai et al. 2000; Romand et al. 2011; Kroon et al. 2019). A good representative for these developmental changes is the visual cortex as it maturates gradually with a sudden increase in sensory input at eye-opening (Lu and Constantine-Paton 2004). In mice, eye opening occurs in 1–2 days, between postnatal day p12 and p14 (Gandhi et al. 2005; Hoy and Niell 2015). Eye opening followed with visual experience shapes not only cortical circuits during critical periods but also retinal and subcortical circuits. It drives synaptic transmission, synaptogenesis, formation, and strengthening of cortical synapses as well as changes in neuronal activity (Yoshii et al. 2003; Lu and Constantine-Paton 2004; Maffei et al. 2004; Wallace and Bear 2004; Zhao et al. 2006). After eye opening, a further critical period between 3 and 4 weeks of age determines the development of binocularity (Gordon et al. 1996; Espinosa and Stryker 2012). Given the abrupt changes during the critical periods, it is important that neurons are able to maintain a stable function which they achieve by homeostatic plasticity (Turrigiano et al. 1998; Turrigiano and Nelson 2004). With the increased excitatory inputs resulting from eye-opening, these homeostatic mechanisms allow neurons to adjust their excitability and synaptic weights in a layer-specific manner (Desai et al. 2002; Maffei et al. 2004, 2006; Goel and Lee 2007). Without homeostatic maintenance, optimizing the level of network excitability in order to allow the detection of incoming signals would not be possible, impeding an appropriate output (Davis and Bezprozvanny 2003; Turrigiano and Nelson 2004).

Few studies have so far investigated developmental changes in electrophysiological and morphological properties of cortical neurons. Generally, these properties have been investigated either in juvenile or young adult animals only (Marx and Feldmeyer 2013; Van Aerde and Feldmeyer 2015; Gouwens et al. 2019). A recent study by the Allen Institute already provided a detailed overview of cell types in the different layers of mouse visual cortex, but only young adulthood was explored (Gouwens et al. 2019). Combining genetics, electrophysiology and morphology identified a number of different inhibitory cell types, whereas pyramidal neurons (PNs) formed more homogenous groups in layer 2/3 (L2/3) and layer 5 (L5). Another study in L5 of rat visual cortex reported changes in intrinsic properties of PNs between p11–p15 and p25–p29 (Etherington and Williams 2011), mainly through a decrease in input resistance and a faster membrane time constant. Here, changes in morphology have only been observed but not quantified. A quantification of physiological and morphological changes during development has only been reported in mouse prefrontal cortex. Here, a similar decrease in excitability could be observed together with morphological changes in the first postnatal month, as dendritic lengths as well as dendritic spine densities increased (Kroon et al. 2019). However, there is still a lack of understanding the combined electrophysiological and morphological changes during development in mouse visual cortex. Yet, the mouse visual system has gained importance in systems and cellular neurophysiology (Niell 2015). Also, in-vitro studies are often focused on young or juvenile cortical tissue, whereas in-vivo studies include electrophysiology, imaging, and behavior from adult mice.

Therefore, we have characterized PNs in the superficial and deeper layers of mouse primary visual cortex both electrophysiologically as well as morphologically. We found, as expected, a decrease in neuronal excitability in both layers. In addition, we observed minor changes in the dendritic morphology in both L2/3 and L5 PNs. Our results show the impact of development on the properties of PNs in the visual cortex from around eye-opening to adulthood.

## Materials and methods

### Animals

All C57BL/6 mice were housed under a normal 12-h light/dark cycle. Before experiments, pups were examined for categorization into “before” and “after eye opening.” Mice of both sexes with different age stages postnatal p10-p14 (young), p25-p29 (juvenile), and p60-p70 (adult) were sacrificed for the experiments (animal license 11451A4 approved by the local authority, LANUV). Mice were controlled for eye opening prior to brain dissection.

Eye opening was observed as a gradual change starting between p10 and p14, on average at day 13.2 ± 0.35 (*n* = 18) similar to previous results (Drager 1978; Gordon et al. 1996). In order to restrict eye opening to a defined time point, eyes can be glued shut and artificially opened (Yoshii et al. 2003). To prevent surgical intervention, we decided to pool mice into a young age group around eye opening (p10–14) similar to previous studies in rat visual cortex (Etherington and Williams 2011) and mouse prefrontal cortex (Kroon et al. 2019).

### Brain slice preparation

Animals were anesthetized by inhalation of isoflurane (AbbVie, UK) and immediately decapitated. Coronal slices (300-μm thick) were cut with a Leica vibratome (Leica VT1200s) in a high magnesium, low calcium solution containing (in mM): 125 NaCl, 2.5 KCl, 1.25 NaH_2_PO_4_, 25 Glucose, 6 MgCl_2_, 1 CaCl_2_, pH 7.4 (95% O_2_/5% CO_2_ and 310 mOsm/l). Slices were incubated at 34 °C for 30 min in artificial cerebrospinal fluid (ACSF) solution containing (in mM): 125 NaCl, 2.5 KCl, 1.25 NaH_2_PO_4_, 25 Glucose, 1 MgCl_2_, 2 CaCl_2_, and pH 7.4 (95% O_2_/5% CO_2_ and ~310 mOsm/l), before being stored at room temperature.

### Whole-cell patch-clamp recordings

Slices were transferred to a recording chamber implanted under an upright microscope (LNscope, Luigs & Neumann, Germany). Slices were superfused with heated ACSF at 30–34 °C. To find the region of interest, a 4× objective (Olympus, Japan) was used and neurons were visualized with a 40× water immersion objective (Zeiss, Germany) using infrared-Dodt (Luigs & Neumann, Germany) gradient contrast with a CMOS camera (Chameleon USB 3.0 monochrome Camera, Point Gray, Canada). Patch pipettes (5–8 MΩ) were pulled from borosilicate glass (GB150F-10, Scientific Products GmbH, Germany) with a horizontal puller (P-1000, Sutter Instruments, Novato, CA, USA) and filled with internal solution containing (in mM): 100 K-gluconate, 20 KCl, 10 Hepes, 4 Mg-ATP, 0.3 Na-GTP, 10 Na_2_-phosphocreatine, pH 7.2 (~300 mosm/l). For the morphology, neurons were filled with the same internal solution containing 0.3% biocytine, Patch-clamp recordings were performed using an ELC-03-XS amplifier (npi electronics, Germany) connected to a Windows-based computer (Dell, Windows 8) with a data acquisition board (PCle 6323, National Instruments, USA). Data were digitized at 20 kHz after lowpass filtering at 10 kHz and acquired with Wavesurfer (version 0.938, Janelia, Ashburn, USA, https://wavesurfer.janelia.org/). Access resistance and electrode capacitance were compensated manually.

PNs were recorded in a whole-cell current-clamp mode and a subset of each age group was filled with biocytin and reconstructed. Cells, which showed no stable resting membrane potential (RMP) or with a high access resistance (*R_a_* > 30 MΩ), were excluded from the analysis. In some cases, incomplete recordings were included in the analysis. Recordings were performed from either layer 2/3 or layer 5 PNs in the mouse primary visual cortex at the different age stages.

Whole-cell patch-clamp recordings consisted of current steps from −100 to 300 pA in steps of 50 pA for 500 ms. The input resistance was calculated as the slope of the current–voltage relationship from −50 to 0 pA current injections. RMP was measured at a current injection of 0 pA. Membrane time constant was calculated with a mono-exponential fit to membrane voltage changes during current steps of −50 pA. The voltage sag was calculated as the percentage difference between the initial voltage response and the sustained voltage response to a current injection of −100 and −50 pA. Afterward, the two responses were linearly fitted and interpolated to a response which would cause a hyperpolarization of −7.5 mV (Van Aerde and Feldmeyer 2015). All active properties were calculated based on the first elicited AP. Spike-frequency adaptation was calculated from at least 10 elicited APs by dividing the first interspike interval by the ninth interspike interval (Marx and Feldmeyer 2013; Van Aerde and Feldmeyer 2015). Stimulus trains were averaged from 10 trials.

### Confocal microscopy and morphology reconstruction

Slices with biocytin-filled neurons were fixed in 4% paraformaldehyde (Sigma-Aldrich, ref: HT501128-4 L) and stored in the fridge at 4 °C overnight. Slices were first washed, then incubated with Streptavidin AlexaFluor-488 (1:800, Invitrogen) in 1% BSA/0.1% triton for 2.5 h. Slices were washed again and mounted with DAPI Fluoromount-G (SouthernBiotech, Birmingham, AL, USA).

Confocal stacks of each slice were obtained with a Leica TCS SP 2 microscope, a 20× magnification objective (HC PL APO 20×/0.70 CS ∞/0.17/C) and a physical size of 750 μm and 1,024 pixel. Overview pictures were taken with 10× magnification (objective: HC PL APO 10×/0.40 CS ∞/0.17/A) and a pixel resolution of 2,048 by 2,048 pixels.

To count the spines, three 3D image stacks per neuron were taken from dendritic branches (30-μm length) with a pixel resolution of 1,024 × 256 and with the number of sections per stack adjusted to the depth of the imaged volume (8–24 μm based on the dendritic morphology; 14–40 sections with 0.4–0.7 μm step distance).

All stacks were analyzed and reconstructed manually with Fiji ImageJ (version 2.0.0) (Schindelin et al. 2012) on a Windows computer, using the plugin Simple Neurite Tracer (version 3.1.6). Neurons with cut apical dendrites were excluded from the analysis of the apical length but were still included in basal and oblique analysis. Based on the tracings, sholl analyses were done using Fiji Imagej Sholl Analysis (version 4.0.0). Dendritic complexity index (DCI) was also calculated (Lom and Cohen-Cory 1999). Spines were counted manually in the 3D image stacks.

### Experimental design and statistical analysis

All values are given as mean values ± standard error of the mean (SEM) if not indicated otherwise. In the violin plots, red lines correspond to the mean values, while black lines correspond to median values. Statistical tests were performed with Matlab (version 2018b, Mathworks). First, a Kolmogorov–Smirnov-Test was used revealing a nonnormal distribution of the data, hence a Wilcoxon test, followed by Bonferroni correction, was performed to test statistically significant differences between data from different ages. Significance levels are given as ^*^ < 0.016, ^*^^*^ < 0.003, and ^*^^*^^*^ < 0.0003 and are displayed in the figures and table.

## Results

### Passive properties

**Fig. 1:**
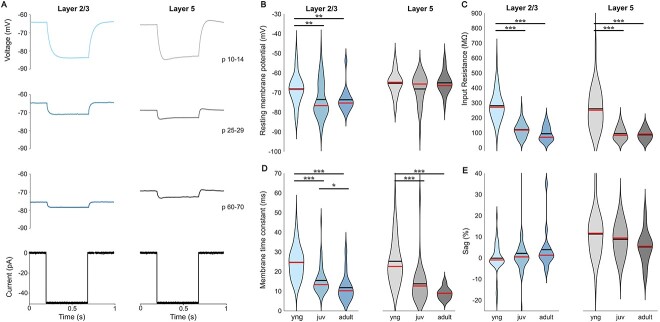
Change of intrinsic membrane properties during development. (A) Examples of voltage traces in response to hyperpolarising current injections of—50 pA for PNs in the different age groups and layers of mouse visual cortex. (B) RMP hyperpolarizes during first month in L2/3 cells. No differences were observed in L5 cells. (C) Input resistance decreases in both layers after eye opening during first postnatal month. (D) Membrane time constant is getting significantly faster in both layers. (E) No significant voltage sag was observed in L2/3 and shows no difference during development in L5.

**Fig. 2:**
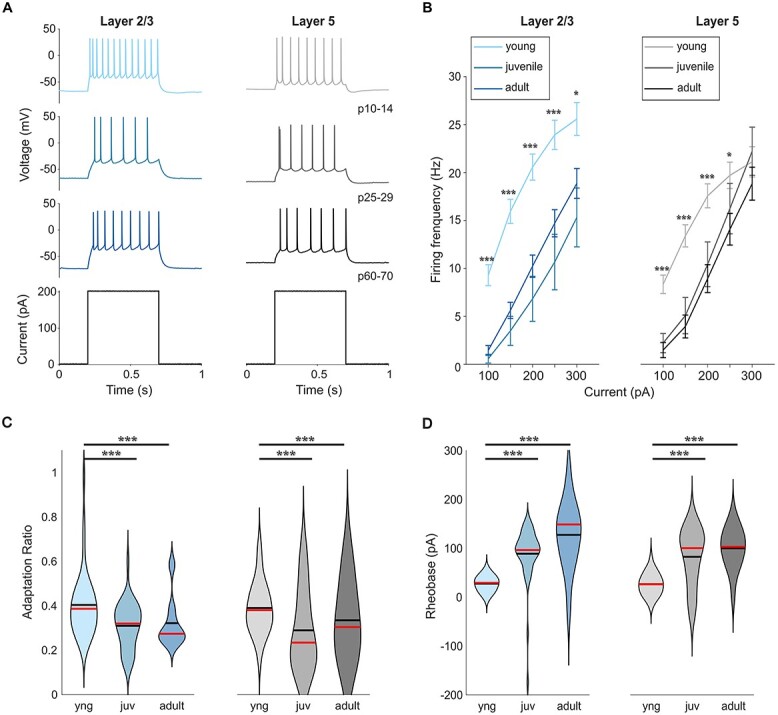
Change of AP firing properties during development. (A) Examples of voltage traces in response to positive current steps of 200 pA in all three age groups and both layers in mouse visual cortex. (B) Firing frequency–current relationship curves show a developmental decrease frequency for the same current injected. (C) Adaptation of firing rate is reduced after eye opening resulting in more sustained AP firing in both layers after first postnatal month.

In this study, a total of 203 PNs were recorded, out of which 119 were from L2/3 and 84 from L5. Cells of different age stages showed different membrane voltage changes in response to hyperpolarizing (Fig. 1A) and depolarizing current injections (Fig. 2A). We first looked at the passive properties (Table 1). We observed that the RMP in L2/3 PNs gets more hyperpolarized during the first postnatal weeks [from −68.1 ± 1.1 mV in p10–14 (young) to −73.5 ± 1.3 mV in p25–29 (juvenile), *P* < 0.003] and remains stable in adult mice (−73.6 ± 1.6 mV in p60–70) (Table 1, Figs. 1B and S1A), while L5 PNs show no significant changes in RMP between young (−65.3 ± 1.0 mV), juvenile (−68.1 ± 1.4 mV), and adult mice (−65.0 ± 1.4 mV) (Table 1, Figs. 1B and S1B).

**Table 1:** Electrophysiological parameters of PNs. Significance level in p10–14 column is resulting from testing it against p25–29; in p25–29 is resulting from testing it against p60–70; in p60–70 is resulting from testing it against p10–14.

	Layer 2/3			Layer 5		
	P10–14	P25–29	P60–70	P10–14	P25–29	P60–70
*Passive properties*						
RMP (mV)	−68.1 ± 1.1 (*n* = 52) ^*^^*^	−73.5 ± 1.3 (*n* = 52)	−73.6 ± 1.6 (*n* = 15) ^*^^*^	−65.3 ± 1.0 (*n* = 36)	−68.1 ± 1.4 (*n* = 29)	−65.0 ± 1.4 (*n* = 15)
*R*_in_ (MΩ)	281.5 ± 13.8 (*n* = 51) ^*^^*^^*^	121.1 ± 7.0 (*n* = 52)	94.0 ± 12.2 (*n* = 15) ^*^^*^^*^	260.8 ± 21.9 (*n* = 36) ^*^^*^^*^	94.4 ± 8.0 (*n* = 29)	86.8 ± 9.4 (*n* = 15) ^*^^*^^*^
τ_c_ (ms)	24.7 ± 1.1 (*n* = 50) ^*^^*^^*^	15.5 ± 1.0 (*n* = 50) ^*^	11.8 ± 1.8 (*n* = 15) ^*^^*^^*^	25.2 ± 1.9 (*n* = 36) ^*^^*^^*^	13.8 ± 1.9 (*n* = 29)	9.0 ± 0.7 (*n* = 15) ^*^^*^^*^
Sag (%)	−0.1 ± 1.1 (*n* = 44)	2.2 ± 1.6 (*n* = 52)	4.0 ± 2.5 (*n* = 15)	11.3 ± 1.7 (*n* = 35)	8.8 ± 1.4 (*n* = 29)	5.3 ± 2.1 (*n* = 15)
*Active properties*						
Firing frequency at 100 pA (Hz)	9.5 ± 1.1 (*n* = 50) ^*^^*^^*^	1.5 ± 0.5 (*n* = 52)	0.6 ± 0.6 (*n* = 15) ^*^^*^^*^	8.3 ± 1.0 (*n* = 36) ^*^^*^^*^	1.5 ± 0.8 (*n* = 29)	1.2 ± 0.8 (*n* = 15) ^*^^*^^*^
Firing frequency at 150 pA (Hz)	16.3 ± 1.2 (*n* = 50) ^*^^*^^*^	5.6 ± 0.8 (*n* = 52)	2.5 ± 1.4 (*n* = 15)	13.4 ± 1.2 (*n* = 36) ^*^^*^^*^	3.9 ± 1.2 (*n* = 29)	3.3 ± 1.8 (*n* = 15) ^*^^*^
Firing frequency at 200 pA (Hz)	21.0 ± 1.3 (*n* = 50) ^*^^*^^*^	10.2 ± 1.2 (*n* = 52)	4.7 ± 2.0 (*n* = 15) ^*^^*^^*^	17.6 ± 1.3 (*n* = 36) ^*^^*^^*^	8.9 ± 1.5 (*n* = 29)	8.4 ± 2.4 (*n* = 15) ^*^
Firing frequency at 250 pA (Hz)	24.4 ± 1.5 (*n* = 50) ^*^^*^^*^	14.7 ± 1.4 (*n* = 52)	7.6 ± 2.5 (*n* = 15) ^*^^*^^*^	19.7 ± 1.4 (*n* = 36) ^*^	14.1 ± 1.6 (*n* = 29)	14.4 ± 2.8 (*n* = 15)
Firing frequency at 300 pA (Hz)	26.1 ± 1.7 (*n* = 50) ^*^	18.9 ± 1.6 (*n* = 52)	12.0 ± 2.7 (*n* = 15) ^*^	21.1 ± 1.6 (*n* = 36)	18.8 ± 1.7 (*n* = 29)	20.7 ± 2.8 (*n* = 15)
Rheobase (pA)	27.6 ± 2.3 (*n* = 50) ^*^^*^^*^	88.8 ± 8.5 (*n* = 44)	127.4 ± 18.3 (*n* = 13) ^*^^*^^*^	26.5 ± 5.7 (*n* = 36) ^*^^*^^*^	82.4 ± 11.1 (*n* = 27)	99.8 ± 10.6 (*n* = 15) ^*^^*^^*^
Gain (Hz/nA)	107.2 ± 4.3 (*n* = 50)	104.8 ± 5.7 (*n* = 44)	118.8 ± 15.0 (*n* = 13)	92.2 ± 5.1 (*n* = 36)	74.5 ± 14.6 (*n* = 27)	82.1 ± 14.3 (*n* = 15)
ISI_1_/ISI_9_	0.40 ± 0.02 (*n* = 45) ^*^^*^^*^	0.31 ± 0.02 (*n* = 37)	0.32 ± 0.04 (*n* = 8) ^*^^*^^*^	0.39 ± 0.02 (*n* = 28) ^*^^*^^*^	0.29 ± 0.04 (*n* = 20)	0.33 ± 0.06 (*n* = 10) ^*^^*^^*^
*AP properties*						
Threshold (mV)	−39.5 ± 0.7 (*n* = 51)	−37.8 ± 0.6 (*n* = 49) ^*^	−34.2 ± 1.2 (*n* = 15) ^*^^*^	−40.0 ± 1.0 (*n* = 36)	−41.5 ± 1.2 (*n* = 27)	−39.5 ± 1.4 (*n* = 15)
Amplitude (mV)	68.4 ± 1.6 (*n* = 51)	69.1 ± 2.0 (*n* = 49)	63.6 ± 3.5 (*n* = 15)	73.0 ± 1.5 (*n* = 36)	77.1 ± 2.0 (*n* = 27)	69.7 ± 3.1 (*n* = 15)
Half width (ms)	1.53 ± 0.04 (*n* = 51) ^*^	1.46 ± 0.10 (*n* = 49)	1.33 ± 0.08 (*n* = 15)	1.77 ± 0.08 (*n* = 36) ^*^^*^^*^	1.07 ± 0.06 (*n* = 27)	1.08 ± 0.09 (*n* = 15) ^*^^*^^*^

Regarding input resistance (*R*_in_), we observed a strong decrease in L2/3 from young (281.5 ± 13.8 MΩ) to juvenile neurons (121.1 ± 7.0 MΩ, *P* < 0.0003), and also in L5 (young: 260.6 ± 21.9 MΩ; juvenile: 94.4 ± 8.0 MΩ, *P* < 0.0003), remaining stable in both layers for adult mice (94.0 ± 12.2 MΩ for L2/3 and 86.8 ± 9.4 MΩ for L5) (Table 1, Figs. 1C and S1C and D). Similarly, membrane time constant (τ_c_) becomes faster during the first postnatal weeks with the strongest change between young (24.7 ± 1.1 ms for L2/3 and 25.2 ± 1.9 ms for L5) and juvenile mice after eye opening (15.5 ± 1.0 ms for L2/3 and 13.8 ± 1.9 ms for L5, *P* < 0.003 for both layers) and it does not significantly change within the second postnatal month (11.8 ± 1.8 ms for L2/3 and 9.0 ± 0.7 ms for L5) (Table 1, Figs. 1D and S1E and F). After pooling these two parameters together across all age groups, we observed a small correlation between *R*_in_ and τ_c_ in L2/3 PNs (*r*^2^ = 0.62, *P* < 0.0001) compatible with the developmental decline of neuronal excitability leading to a decrease in *R*_in_ and an acceleration of τ_c_. The correlation is stronger in L5 PNs (*r*^2^ = 0.78, *P* < 0.0001) and similar to what has been observed in rat L5 PNs (Etherington and Williams 2011).

Finally, we measured the voltage sag. No or only very little sag was observed in L2/3 PNs (Table 1, Fig. 1A and E) across all age groups. In L5 PNs, the sag intensity remained small and stable in young (11.3 ± 1.7%), juvenile (8.8 ± 1.4%), and adult mice (5.3 ± 2.1%), suggesting that the density of hyperpolarization-activated cyclic nucleotide-gated channels does not change during development in the mouse visual cortex.

Overall, these data show that development alters the passive properties of PNs in the visual cortex, leading to a general decrease of their excitability.

### Firing properties

While passive properties changed during development, we also observed differences in active properties and action potential (AP) firing of PNs in both layers. Current steps of different amplitudes were injected to obtain a firing rate to input current relationship (F–I curve) (Fig. 2A and B) to evaluate the active properties of PNs in both layers during development. Comparing firing rates evoked by 200-pA current steps resolved a decrease from young (21.0 ± 1.3 Hz in L2/3 and 17.6 ± 1.3 Hz in L5) to juvenile (10.2 ± 1.2 Hz in L2/3 and 8.9 ± 1.5 Hz in L5) and remained unchanged to adult (7.6 ± 2.5 Hz in L2/3 and 14.4 ± 2.8 in L5) (Table 1, Fig. 2B). Also, the adaptation ratio decreased from young to juvenile PNs in both layers (Table 1, Fig. 2C). We did not observe a significant difference in the firing rates at 300 pA in L5, due to a depolarization block occurring in some young PNs at 250 pA and above. From the F–I curve, we obtained an estimated gain and rheobase from a linear regression between 100 and 200 pA. While we did not see a change in the gain, there is a significant shift in the rheobase from young (27.6 ± 2.3 pA in L2/3 and 26.5 ± 3.8 pA in L5) to juvenile (88.8 ± 8.5 pA in L2/3 and 82.4 ± 11.1 pA in L5). There is no significant shift from juvenile to adult (127.4 ± 18.3 pA in L2/3 and 99.8 ± 10.6 pA in L5) (Table 1). These data indicate that the reduced firing rates are the consequence of a developmental shift of the rheobase.

**Fig. 3:**
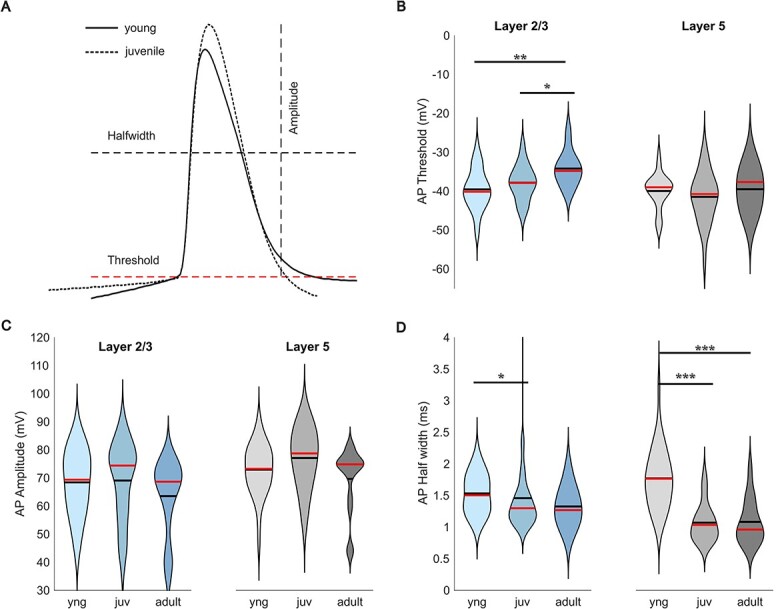
Change of AP properties during development. (A) Example of an AP from a young and juvenile L2/3 PN indicating analyzed parameters. (B) AP threshold increased in L2/3, no change was observed in L5. (C) AP amplitudes showed no significant differences during development in both layers. (D) APs became faster with reduced half widths in both layers after eye-opening.

By eliciting a single AP (Fig. 3A), we analyzed three of its parameters: threshold, amplitude, and half-width. The AP threshold increased gradually with age in layer 2/3 PNs (−39.5 ± 0.7 mV in young, −37.8 ± 0.6 mV in juvenile, and −34.2 ± 1.2 mV in adult) and showed no difference in layer 5 PNs (−40.0 ± 0.8 mV in young, −41.5 ± 1.2 mV in juvenile, and −39.5 ± 1.4 mV in adult) (Table 1, Fig. 3B). We also observed a developmental decrease of AP half-width mainly between young and juvenile in L2/3 PNs (1.53 ± 0.04 vs 1.46 ± 0.10 ms) and in L5 PNs (1.77 ± 0.08 vs 1.07 ± 0.08 ms in juvenile) (Table 1, Fig. 3D), but there is no significant effect of development on AP amplitude in both layers (Table 1, Fig. 3C). These data show that PNs are less excitable with evoked AP firing rates becoming lower with stronger adaption after eye opening.

### Morphology

**Fig. 4:**
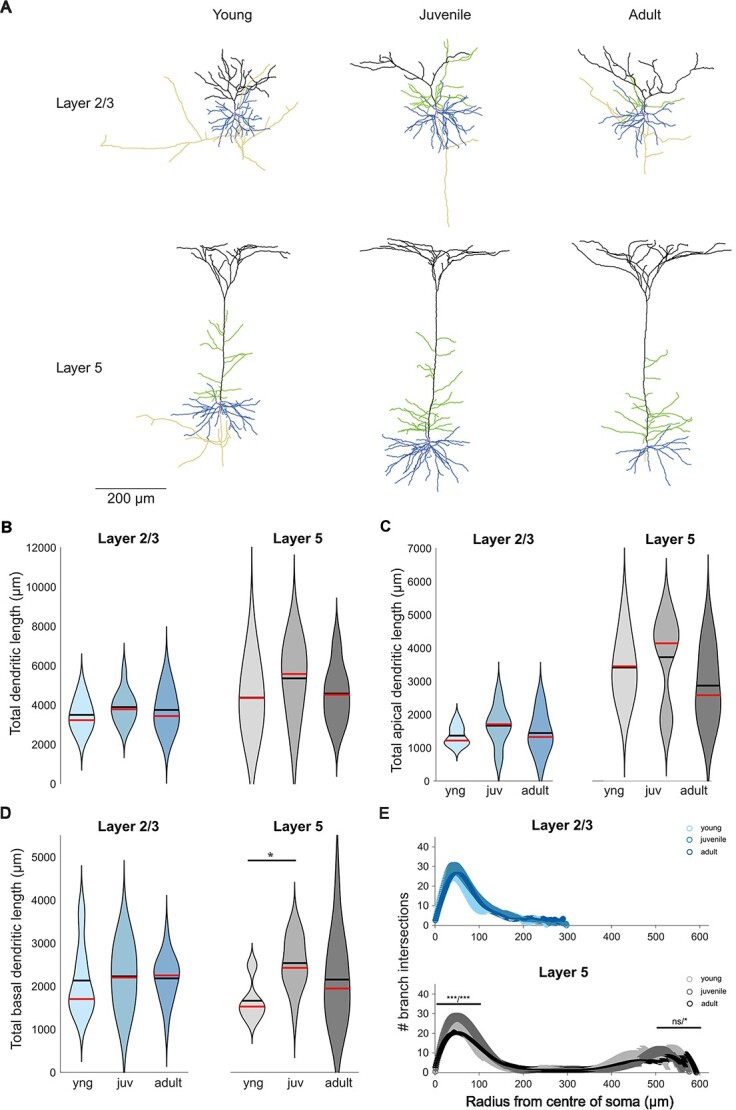
Developmental changes in dendritic morphology. (A) Example of reconstructed L2/3 and L5 PNs from all three age stages. (B) Total dendritic length does not change in both layers. (C) Total apical dendritic length does not change in both layers. (D) Total basal dendritic length increases during the first postnatal month in L5 PNs only. (E) Sholl analysis shows no developmental influence on PN morphology in L2/3, while there is a significant change in L5 PNs the radius closer to the soma (^*^^*^^*^ when comparing young to juvenile and ^*^^*^^*^ when comparing juvenile to adult). Above 500 μm from the soma to the end, there is no significant change when comparing young to juvenile, but there is a significant difference when comparing juvenile to adult (^*^).

Filling the recorded neurons with biocytin, we reconstructed a total of 71 PNs in both layers of the different age groups. For the morphology analysis, only neurons with complete apical dendrites and no signs of severing dendritic arbors or tufts were included (Figs. 4A, S2, and S3). Overall, there was no developmental change in the morphology and dendritic complexity of L2/3 PNs (Fig. 4, Table 2). In L5, we also observed no change in the total dendritic length and dendritic complexity (Fig. 4B, Table 2), but in-depth analysis revealed an increase in the basal dendritic length (1,660.7 ± 146.1 μm in young, 2,536.4 ± 167.8 μm in juvenile, and 2,261.2 ± 261.4 μm in adult; Fig. 4D and Table 2). Sholl analysis showed no significant impact of development on L2/3 PN morphology (Fig. 4E), while there was a significant decrease for L5 PNs in number of branch intersections at proximal dendrites (below 100 μm from the soma, *P* < 0.0003 for young vs juvenile and juvenile vs adult, *P* < 0.0003 for adult vs young) and distal (further than 500 μm from the soma, *P* > 0.016 for young vs juvenile, *P* < 0.016 for juvenile vs adult, *P* < 0.0003 for adult vs young) (Table 2). These data indicate that eye-opening does not change the overall morphology of L2/3 PNs and that L2/3 PNs dendrites already reached their full size at this young age. For L5 PNs, significant changes and growth processes were visible in the analyzed time course.

**Table 2:** Morphological parameters of PNs. Sample size in dendritic size corresponds to individual neurons, while sample size in spine density corresponds to individual neurites. Significance level in p10–14 column is resulting from testing it against p25–29; in p25–29 is resulting from testing it against p60–70; in p60–70 is resulting from testing it against p10–14.

	Layer 2/3			Layer 5		
	P10–14	P25–29	P60–70	P10–14	P25–29	P60–70
*Dendritic size*						
Apical length (μm)	1364.6 ± 112.5 (*n* = 7)	1664.8 ± 171.9 (*n* = 12)	1444.6 ± 154.6 (*n* = 16)	3411.6 ± 354.5 (*n* = 7)	3724.2 ± 516.6 (*n* = 7)	2868.4 ± 328.4 (*n* = 10)
Basal length (μm)	2131.2 ± 309.9 (*n* = 7)	2227.7 ± 195.4 (*n* = 12)	2181.6 ± 129.2 (*n* = 16)	1660.7 ± 146.1 (*n* = 10) ^*^	2536.4 ± 167.8 (*n* = 12)	2152.5 ± 265.3 (*n* = 14)
Oblique length (μm)	322.1 ± 70.0 (*n* = 7)	607.9 ± 159.8 (*n* = 11)	444.1 ± 66.7 (*n* = 16)	1121.4 ± 123.5 (*n* = 10)	1190.2 ± 147.5 (*n* = 12)	951.2 ± 117.8 (*n* = 14)
Total dendritic length (μm)	3495.8 ± 290.6 (*n* = 7)	3892.5 ± 236.1 (*n* = 12)	3746.2 ± 250.2 (*n* = 16)	4372.0 ± 542.0 (*n* = 10)	5352.9 ± 508.1 (*n* = 12)	4576.9 ± 377.7 (*n* = 14)
Proximal branch intersections (< 100 μm)	20.26 ± 0.70 (*n* = 7)	20.97 ± 0.70 (*n* = 12)	19.40 ± 0.64 (*n* = 16)	8.30 ± 0.37 (*n* = 10) ^*^^*^^*^	7.22 ± 0.38 (*n* = 12) ^*^^*^^*^	6.02 ± 0.23 (*n* = 14) ^*^
Distal branch intersections (>500 μm)	-	-	-	17.91 ± 0.59 (*n* = 10)	22.05 ± 0.65 (*n* = 12) ^*^	16.46 ± 0.43 (*n* = 14) ^*^^*^^*^
Total branch points	56.1 ± 4.5 (*n* = 7) ^*^	41.2 ± 3.2 (*n* = 12)	42.7 ± 2.3 (*n* = 16) ^*^	53.4 ± 6.4 (*n* = 10)	53.0 ± 5.5 (*n* = 12)	40.6 ± 3.7 (*n* = 14)
DCI (×10^3^)	131.8 ± 25.3 (*n* = 7)	135.7 ± 19.7 (*n* = 12)	109.3 ± 13.4 (*n* = 16)	198.1 ± 32.4 (*n* = 7)	245.8 ± 46.7 (*n* = 7)	141.5 ± 20.8 (*n* = 10)
*Spine density (spines/μm)*						
Apical	0.59 ± 0.07 (*n* = 15) ^*^^*^^*^	1.08 ± 0.08 (*n* = 30)	0.90 ± 0.05 (*n* = 43) ^*^^*^^*^	0.42 ± 0.04 (*n* = 17)	0.52 ± 0.05 (*n* = 11)	0.40 ± 0.03 (*n* = 25)
Basal	0.53 ± 0.08 (*n* = 14) ^*^^*^^*^	0.93 ± 0.07 (*n* = 27)	0.87 ± 0.04 (*n* = 34) ^*^^*^^*^	0.54 ± 0.03 (*n* = 22)	0.56 ± 0.05 (*n* = 16)	0.59 ± 0.05 (*n* = 18)
Total	0.56 ± 0.05 (*n* = 29) ^*^^*^^*^	1.01 ± 0.05 (*n* = 57)	0.88 ± 0.03 (*n* = 77) ^*^^*^^*^	0.49 ± 0.03 (*n* = 39)	0.54 ± 0.04 (*n* = 27)	0.48 ± 0.03 (*n* = 43)

**Fig. 5:**
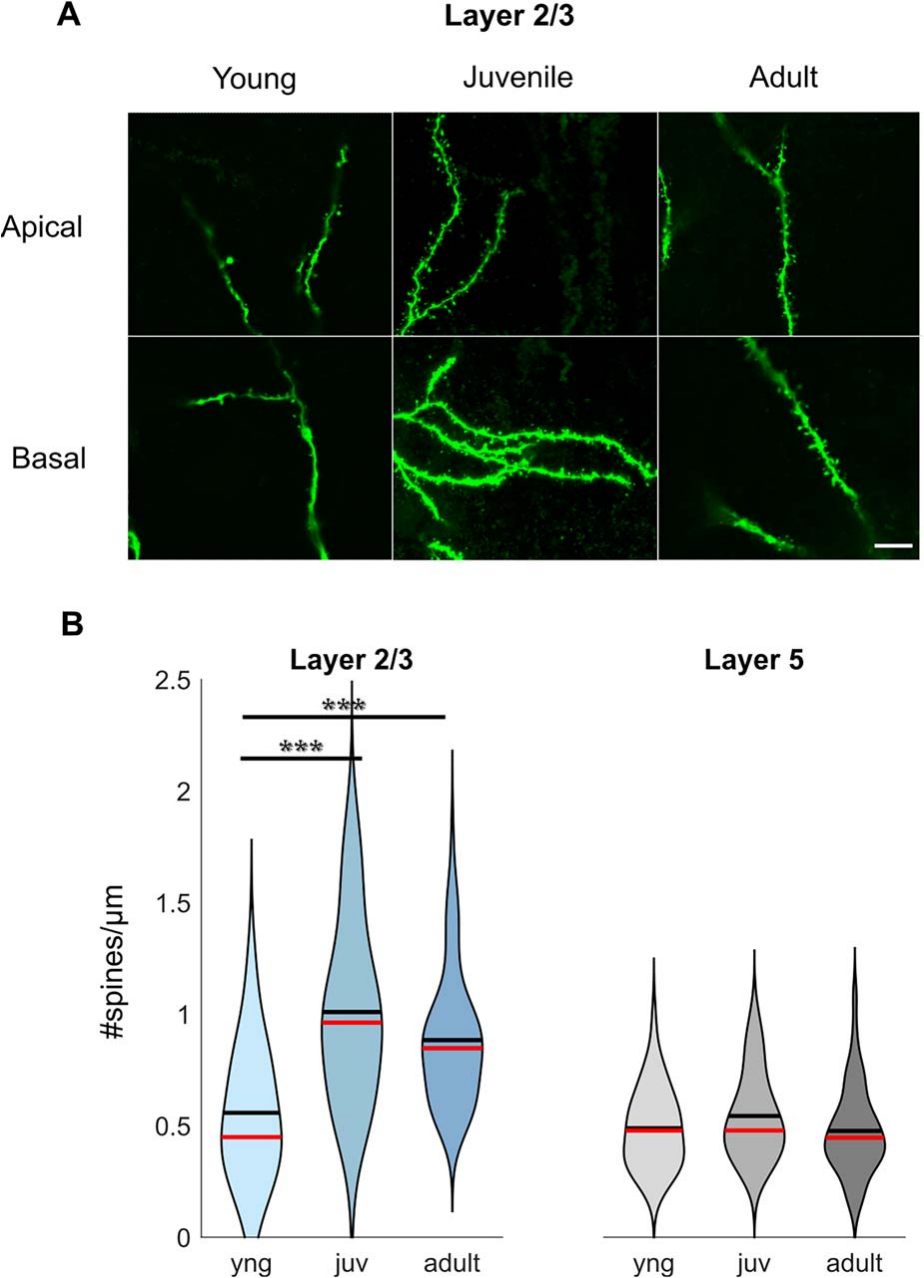
Developmental changes in spine density. (A) Confocal images of apical and basal dendrites with spines from L2/3 PNs from all age groups. Scale bar = 8 μm. (B) Spine density only increases in L2/3 PNs during first postnatal month.

Next, we calculated the spine density of apical and basal dendrites (Fig. 5A), separately (Table 2), and pooled together (Fig. 5B and Table 2). In L2/3 PNs, spine density increases drastically during the first postnatal month, from young (0.59 ± 0.07 spines/μm in apical dendrites, 0.53 ± 0.08 spines/μm in basal dendrites, and 0.54 ± 0.05 spines/μm in total) to juvenile (1.08 ± 0.08 spines/μm in apical dendrites, 0.93 ± 0.07 spines/μm in basal dendrites, and 1.01 ± 0.05 spines/μm in total), and it remains stable during adulthood (0.90 ± 0.05 spines/μm in apical dendrites, 0.87 ± 0.04 spines/μm in basal dendrites, and 0.88 ± 0.03 spines/μm in total) (Fig. 5B, Table 2). However, there is no developmental change in spine density in L5 PNs (Fig. 5B, Table 2).

Altogether, the observed changes in PN morphology show minor growth in dendritic length or branches. However, we observed a 2-fold increase in synaptic spines after eye opening in superficial L2/3.

## Discussion

In this study, we investigated developmental changes of visual cortex L2/3 and L5 PNs around the time of eye opening and the critical period. Most changes occur in the first postnatal month, around the time of eye opening, when visual input is introduced. At this time point, we found a decrease in *R*_in_ correlating with faster membrane time constants in both layers. The RMP becomes more hyperpolarized, but only in L2/3 neurons. Looking into AP firing properties confirmed the reduced excitability after eye opening with lower evoked firing rates in response to prolonged current injections and stronger firing rate adaption in both layers. AP threshold is increased after eye opening. AP half-width and rise-time decreased mainly in L5 neurons with age. In addition to changes in electrophysiological properties during visual cortex development, we investigated changes in dendritic morphology with age. While spine density nearly doubled after eye opening between young and juvenile L2/3 PNs, dendritic length did not increase with the exception of basal dendrites in L5 PNs. In general, neurons become less excitable after eye opening as observed by more negative RMPs, a higher firing threshold, higher rheobase, and reduced firing rates in response to prolonged current steps. Also, neurons in L2/3 increase their spine density with development. These changes are presumably driven by eye opening leading to an increase in synaptic input, as it has been observed in the rat visual cortex (Tatti et al. 2017). Therefore, more spines are formed and stabilized, while homeostatic mechanisms counterbalance the increased synaptic input with a reduced excitability of the neurons.

Looking into the morphological properties, it has been shown that cortical PNs undergo developmental changes in dendritic morphology (Petit et al. 1988; Nuñez-Abades et al. 1994; Franceschetti et al. 1998; Zhang 2004; Romand et al. 2011; Kroon et al. 2019). Our results indicate that maturation of PN morphology in visual cortex occurs before eye-opening and independent of visual experience similar to previous reports (Miller 1981; Richards et al. 2020). We find a subtle remodeling of L5 basal dendrites after eye opening, which might reflect adaptations in synaptic connectivity between neighboring PNs at juvenile age (Frick et al. 2007; Etherington and Williams 2011).

Our measured spine densities match with findings from other groups in other cortical areas, showing L2/3 PNs possessing higher densities than L5 PNs (Virtanen et al. 2018; Kroon et al. 2019). Spine density remained stable into adulthood, as it was observed by others (Grutzendler et al. 2002). Our data in mouse visual cortex show a developmental increase in the spine density only in L2/3 (Fig. 5B). In rat visual cortex, an increase in spontaneous excitatory postsynaptic current frequency has been observed in L2/3 PNs but not in L5 PNs (Tatti et al. 2017), which could be explained by the increase in spine density, as the frequency of synaptic inputs is correlated with the number of release sites and synapses (De Roo et al. 2008).

Comparable age-related changes in intrinsic properties have been observed in other cortical areas, as rat somatosensory cortex (Frick et al. 2007), rat auditory cortex (Oswald and Reyes 2008), rat motor cortex (Perez-García et al. 2021), mouse medial prefrontal cortex (Kroon et al. 2019), as well as rat primary visual cortex (Etherington and Williams 2011; Tatti et al. 2017). During maturation, the membrane potential becomes more hyperpolarized, while the input resistance decreases. These intrinsic changes could be caused by a shortening of the axonal initial segment as shown for the primary visual cortex (Gutzmann et al. 2014). During maturation, the axon initial segment length declines between p15 and p28, which is triggered by the onset of vision. The shortening begins from the distal end. As a result of a shortened axonal initial segment, probably less voltage-gated sodium and potassium channels are distributed on this segment, having an impact on the AP properties as we observed (see Table 1) leading to a decrease in excitability.

A recent study has fully characterized the neuronal population of the mouse adult visual cortex (Gouwens et al. 2019). In general, our data are similar to what has been observed in these studies. The difference might arise from the method of selecting neurons to record, as they used different transgenic mouse lines to emphasize each layer, while we used a nontransgenic C57BL/6 mouse line for both layers. The difference might also come from the difference in sample size and mouse lines. Here, we provided a characterization of intrinsic properties of L2/3 and L5 PNs in mouse visual cortex and the effect of development on its properties.

Regarding PNs heterogeneity, it has been shown in several cortical areas that there are at least two subtypes of L5 PNs (Baker et al. 2018). In visual cortex, three subtypes have been identify based on their passive properties, transcriptomic profile, and projections as cortico-cortical (CC), cortico-subcortical (CS), and cortico-cortical-non-striatal (CC-NS) (Kim et al. 2015). We therefore performed principal component analysis followed by k-means to identify possible clusters within our data set, but we did not find any evidence for these subtypes (Fig. S4). This can be explained by the fact that we blindly selected L5 PNs and that there is a higher proportion of CC (or L5a) PNs within the visual cortex (Kim et al. 2015). To better distinguish them, specific mouse lines (Kim et al. 2015; Gouwens et al. 2019) or retrograde labelling (Brown and Hestrin 2009) would be required. However, since we show here that L2/3 and L5 neurons are similarly impacted by development, it is expected that all L5 PN subtypes experience similar developmental changes.

Changes in neural excitability are intrinsic and begin during the onset of sensory input. In primary visual cortex, at the time of eye-opening, visual input drives changes in intrinsic properties of PNs. In mouse auditory cortex, similar results regarding the decrease of excitability in PNs were reported with the onset of hearing by a change in RMP and resistance, rather than by changes in the AP threshold (Oswald and Reyes 2008).

Similar observations were obtained in vivo (Brown et al. 2019). Visually deprived mice showed a more depolarized RMP and AP threshold, compared with the control group with a normal dark–light cycle (Brown et al. 2019), and are comparable to results obtained before and around the time of eye opening in this study (Brown et al. 2019). Also, ongoing activity appears in dense spontaneous waves in primary visual cortex of mice before eye-opening. After onset of visual experience, however, spontaneous activity becomes sparse (Rochefort et al. 2009), which can be explained by the reduced excitability observed in our study as a result of the increased synaptic input after eye-opening (Rochefort et al. 2009). A parsimonious explanation for the reduced excitability correlating with increased sensory inputs is homeostatic regulation to keep the neuronal activity level constant (Jamann et al. 2021). Homeostatic plasticity can be mediated by voltage-dependent sodium, calcium and potassium conductances, which are displayed in the intrinsic and firing properties of a cell (Turrigiano et al. 1994; Frank 2014; Tien and Kerschensteiner 2018; Rátkai et al. 2021).

Characterizing the effect of development on neuronal properties is crucial as most in-vitro studies used juvenile animals, while there are more in-vivo studies correlating behavior with electrophysiology and imaging in adult animals. Providing comprehensive data from pyramidal cells in L2/3 and L5 in visual cortex from mice around eye opening as well as at juvenile and adult age stages, our study fills the gap in knowledge of the developmental changes in electrophysiology and morphology, from before eye-opening to adulthood.

## Supplementary Material

Ciganok_Jehasse_Supplementary_data_bhac467
