# Supplementary material for: Postnatal development of electrophysiological and morphological properties in layer 2/3 and layer 5 pyramidal neurons in the mouse primary visual cortex
Source: Cereb Cortex. 2022 Dec 1;33(10):5875–84. doi: 10.1093/cercor/bhac467 (PMC10183751; doi:10.1093/cercor/bhac467)
Supplement: Ciganok_Jehasse_Supplementary_data_bhac467 [file ciganok_jehasse_supplementary_data_bhac467.pdf]

Postnatal development of electrophysiological and morphological properties in layer 2/3 and layer 5 pyramidal neurons in the mouse primary visual cortex

Running Title:

Neuronal development in the mouse visual cortex

Natalja Ciganok-Hückels[1,4,#], Kevin Jehasse[1,#], Lena Kricsfalussy-Hrabár[1], Mira Ritter[1], Thomas Rüland[1,2,4], Björn M. Kampa[1,3,4*]

# these authors contributed equally

* Correspondence: Björn M. Kampa, Email: kampa@brain.rwth-aachen.de

Supplemental Data

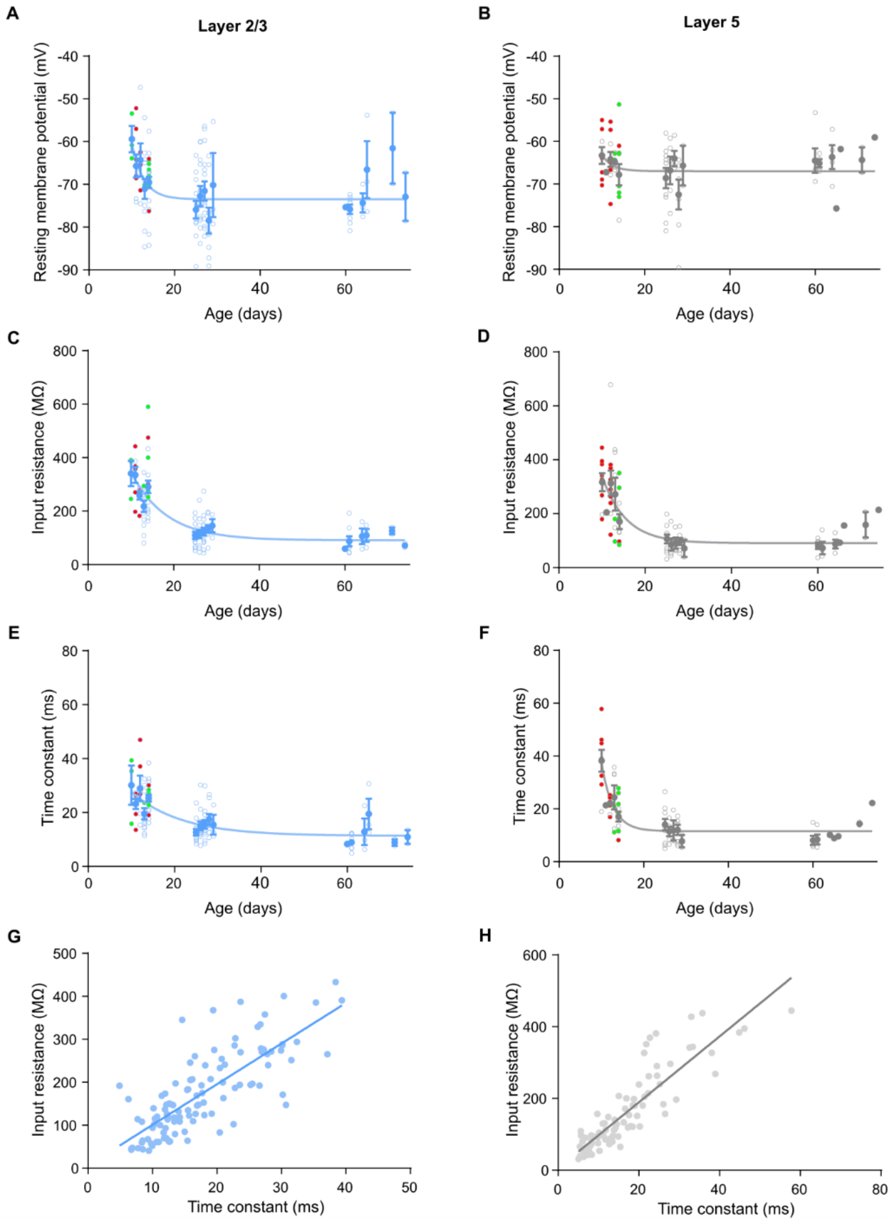

Figure S1. Postnatal development changes of passive properties of L2/3 and L5 PNs. (A-F) Summary graphs reveal age-depend decreases in RMP in L2/3 only (A-B), in Rin in both layers (C-D) and τc in both layers. Connecting lines were obtained from single exponential fits. Light color points correspond to individual data while darker points correspond to the mean with SEM. Red filled circles correspond to data from young mice where the eyes were clearly closed whereas green filled circles indicate correspond to data from young mice where eyes have been already clearly open. (G-H) Positive correlation between Rin and τc in L2/3 (r^2 = 0.62, $p < 0.0001$) and L5 (r^2 = 0.78, $p < 0.0001$) PNs are in favor of a postnatal decline of neuronal excitability.

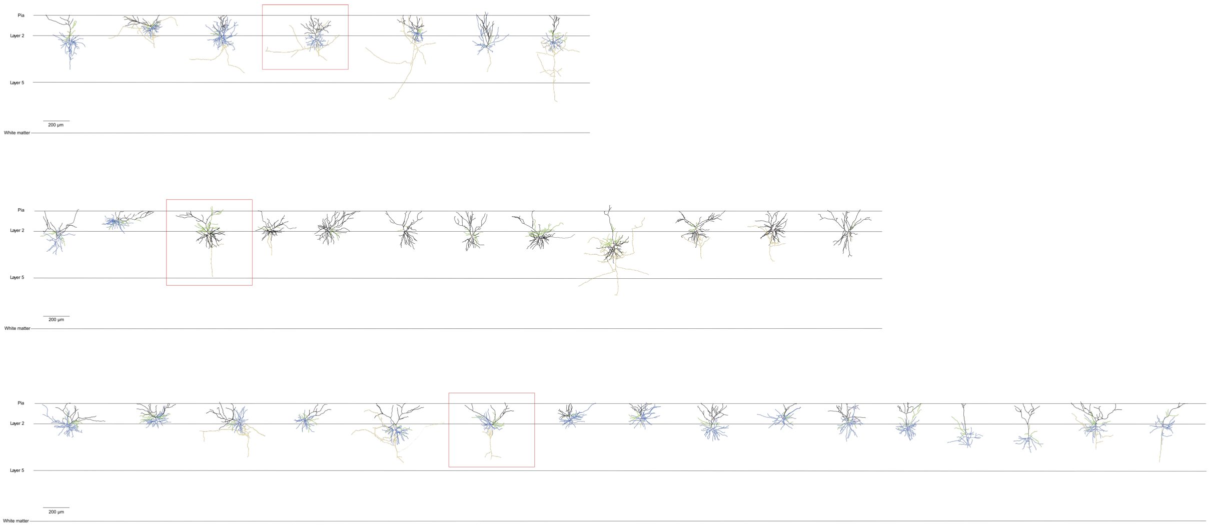

Figure S2. Reconstruction of L2/3 PNs at different age stage. Top panel corresponds to young, middle panel to juvenile and bottom to adult PNs. Black traces correspond to apical, green to oblique and blue to basal dendrites and yellow traces correspond to the axon. Red rectangles correspond to reconstructed used for figure 4A. Scale bar = 200 µm.

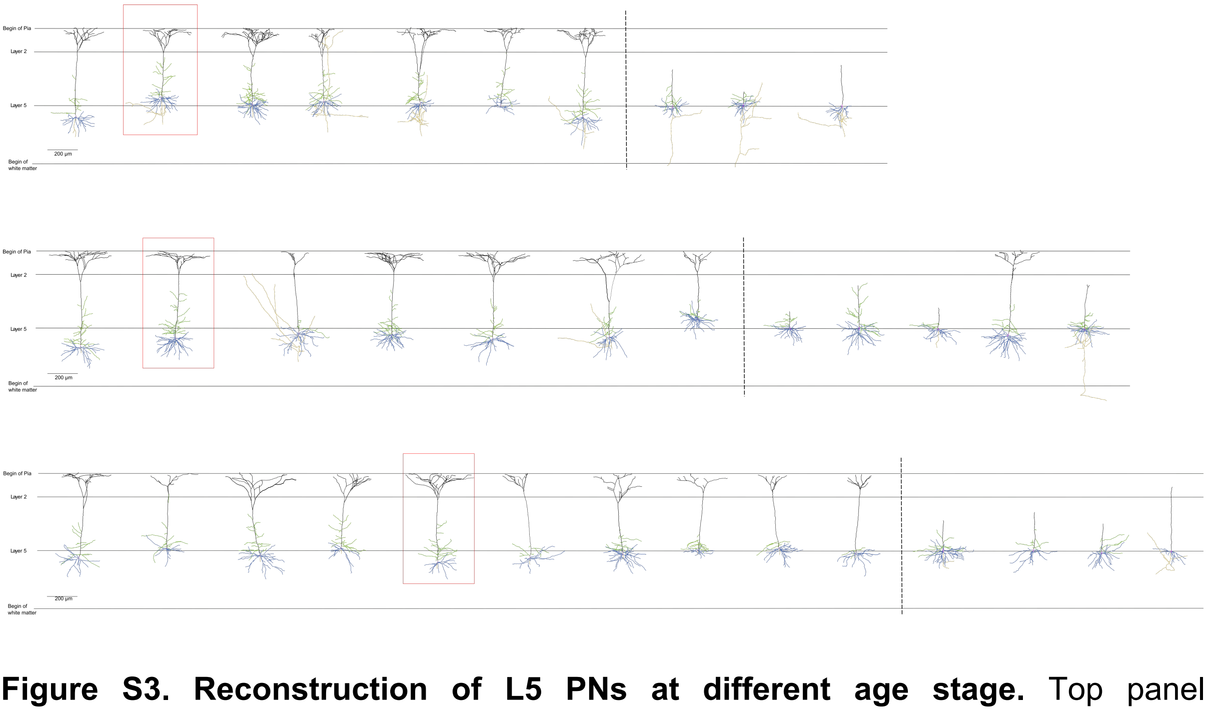

Figure S3. Reconstruction of L5 PNs at different age stage. Top panel corresponds to young, middle panel to juvenile and bottom to adult PNs. Black traces correspond to apical, green to oblique and blue to basal dendrites and yellow traces correspond to the axon. The vertical dashed lines separate the neurons with intact apical dendrites (left) from neurons with cut apical dendrites (right). Red rectangles correspond to reconstructed used for figure 4A. Scale bar = 200 µm.

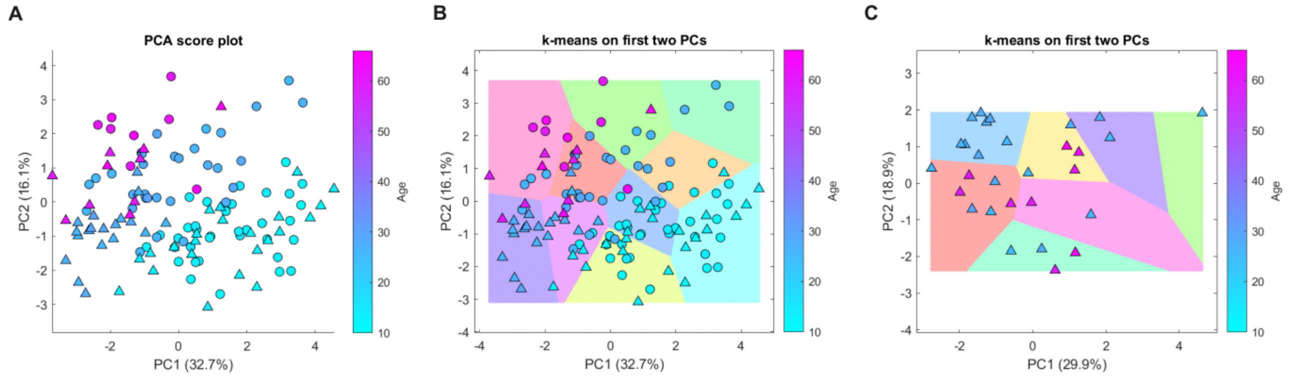

Figure S4. Cluster analysis of L2/3 and L5 PNs. (A) PCA score plot using the first two principal components explaining more than 50% of variability. Circles correspond to L2/3 PNs, triangles correspond to L5 PNs. (B) K-means cluster analysis on PCA score plot from (A) does not reveal clear clusters in the dataset. (C) K-means cluster analysis on PCA scores from juvenile and adult L5 PNs alone also does not reveal any separation of different subtypes.
